# The keratinized mucosa width at partially edentulous molar sites and its associated factors: a pilot study

**DOI:** 10.1186/s12903-022-02669-2

**Published:** 2022-12-22

**Authors:** Ziyao Han, Cui Wang, Yiping Wei, Gang Yang, Wenjie Hu, Kwok-Hung Chung

**Affiliations:** 1 Department of Periodontology, National Clinical Research Center for Oral Disease, National Engineering Research Center of Oral Biomaterials and Digital Medical Devices, Beijing Key Laboratory of Digital Stomatology, Peking University School and Hospital of Stomatology, 22 Zhongguancun S Ave, Haidian District, 100081 Beijing, China; 2 NHC Research Center of Engineering and Technology for Computerized Dentistry, Beijing, China; 3 Department of Restorative Dentistry, University of Washington, Seattle, WA USA

**Keywords:** Keratinized mucosa, Phenotype, Edentulous ridge, Soft tissue assessment

## Abstract

**Background:**

Assessment of the keratinized mucosa width (KMW) at edentulous sites is important for the subsequent implant treatment design. This pilot study aimed to evaluate the characteristics of the KMW at edentulous molar sites and explore the associated factors.

**Methods:**

A total of 150 patients with 222 edentulous molar sites were included. The buccal KMW of the edentulous molar sites was measured during implant treatment planning. Potentially associated factors, including age, sex, smoking status, location, reasons for tooth loss/extraction, gingival phenotype (GP) and keratinized gingival width (KGW) of the adjacent teeth, were collected and analyzed. The Shapiro‒Wilk test, Student’s *t test*, one-way ANOVA, generalized estimation equations (GEEs) and linear regression analysis were used for data analysis at *α* = 0.05.

**Results:**

The buccal KMW at edentulous molar sites was 3.97 ± 2.06 mm, and 41.9% of sites presented with KMW < 4 mm. The mean KMWs of the maxillary sites were significantly higher than that those of the mandibular sites (4.96 ± 2.05 mm vs. 3.41 ± 1.85 mm, respectively). In total, 54.7%, 46.5%, 29.8%, and 0.0% of mandibular first and second molar sites and maxillary first and second molar sites, respectively, displayed a KMW of < 4 mm. Statistically significant linear correlations were found between KMW and GP (r = 0.161, *p* = 0.025) and between KMW and KGW of the adjacent teeth (r = 0.161, *p* = 0.023), while other factors were found to have no significant association.

**Conclusion:**

Within the limitations of the present study, the KMW at edentulous molar site was related to the location of molar tooth loss/extraction. The GP and KGW of the adjacent teeth of edentulous molar sites were also associated with their KMW, which was probably attributed to the continuity of the adjacent soft tissue.

## Background

The keratinized mucosa width (KMW) is the apical-coronal distance from the mucosal margin to the mucogingival junction (MGJ) and constitutes the indispensable component of the peri-implant phenotype [1]. For the rehabilitation of missing molars, the implant treatment has become a popular and predictable choice, and the KMW constitutes the indispensable component of the peri-implant phenotype [1–3]. According to previous studies, 18–74% of dental implants presented with an inadequate KMW (< 2 mm), which would be a risk factor for the development of peri-implant diseases [1, 4–13]. To ensure the implant was surrounded by adequate KMW (≥ 2 mm), it was preferable that an adequate band of keratinized mucosa had already been maintained and existed before implant therapy [14–16].

For edentulous ridges, the definition of an “adequate” KMW may differ from that for peri-implant tissues. Although clinicians try to avoid the sacrifice of KMW during implant procedures, it is oftentimes reduced to a certain extent (mean of 1.36–1.65 mm, at the buccal aspect) due to soft tissue modeling during postoperative wound healing, abutment connection and crown insertion [16–20].Considering the reduction in KMW during implant treatment, having a minimum of 4 mm KMW at the edentulous ridge was predicted to maintain an adequate peri-implant KMW (≥ 2 mm) when implant treatment at the edentulous sites was completed, according to the latest reports [16, 17, 19, 20]. It should be taken into consideration during implant treatment planning whether adequate KMW of the edentulous sites would be available and if soft tissue augmentation should be performed.

Studies focusing on the dimensions of KMW at edentulous sites and the exploration of its associated factors are sparse. Only the characteristics of keratinized gingiva width (KGW) / attached gingiva width (AGW) around natural adjacent teeth and the KMW around future implant sites have been discussed. Regarding the natural teeth, the buccal KGW/AGW is higher at the maxillary teeth than at the opposite mandibular teeth [21–23]. Lang and Löe [22] also reported that the maxillary facial KGW is approximately 1.0 mm wider than that of the mandible. Factors including the position of the tooth, high frenum and muscle attachments, gingival thickness (GT), gingival phenotype (GP), and gingival recession are associated with the AGW around natural teeth [24–28]. In addition, an inadequate KGW is likely to be accompanied by a thin GT and thin GP [24, 27–29].

The peri-implant KMW is higher at maxillary implants than at homonymous mandibular implants. Influencing factors such as implant position, reasons for tooth loss, and bone augmentation procedures have been disclosed [13]. The relationship of peri-implant KMW and the KGW of adjacent teeth was once analyzed [4]. It was reported that when implants are surrounded by a narrow (≤ 2 mm) keratinized mucosa or only by alveolar mucosa, the KGW of adjacent teeth is also narrower [4].

The assessment of KMW at edentulous sites is important for the subsequent implant treatment design. Therefore, the purpose of this pilot study was to assess the dimensional characteristics of the buccal keratinized mucosa (KM) of edentulous molar sites and to explore the potentially associated factors.

## Methods

### Study population, inclusion and exclusion criteria

The present study was retrospectively cross-sectionally designed. Subjects were selected from patients with missing molars attending the Department of Periodontology for implant rehabilitation from June 2013 to October 2020. This study was approved by the Institutional Review Board of Peking University School and Hospital of Stomatology (No. PKUSSIRB-202,058,143) in December 2020 and was performed in accordance with the Helsinki Declaration revised in 2013 and the STROBE guidelines. All the recruited patients signed an informed consent form. The inclusion and exclusion criteria were as follows:

Inclusion criteria: (1) greater than 25 years old (to exclude the influence of erupting third molars on implant treatment); (2) systemically healthy and taking no medications known to increase the risk of gingival enlargement or affect soft tissue healing; (3) at least one molar lost (excluded third molars); (4) periodontally healthy clinically according to the consensus report by Chapple et al. [30].

The exclusion criteria were as follows: (1) incomplete dental records; (2) pregnancy or lactation; (3) history of soft tissue augmentation at the edentulous sites; and (4) history of bisphosphonate use or head and neck radiotherapy.

Records of the recruited subjects and edentulous molar sites were reviewed to collect the following information: (1) general information: age, and sex; smoking status: smoker or nonsmoker; (2) information on the edentulous sites: locations (maxilla or mandible, first or second molar), reasons for tooth loss/extraction: periodontal-related reasons (loss or extraction due to advanced periodontal disease or periodontal-endodontic combined lesions), nonperiodontal related reasons (loss or extraction due to an unsalvageable residual crown and root, endodontic and periapical lesions, crown and root fractures, trauma, or other causes of tooth loss or tooth agenesis).

### Clinical parameters

**Fig. 1:**
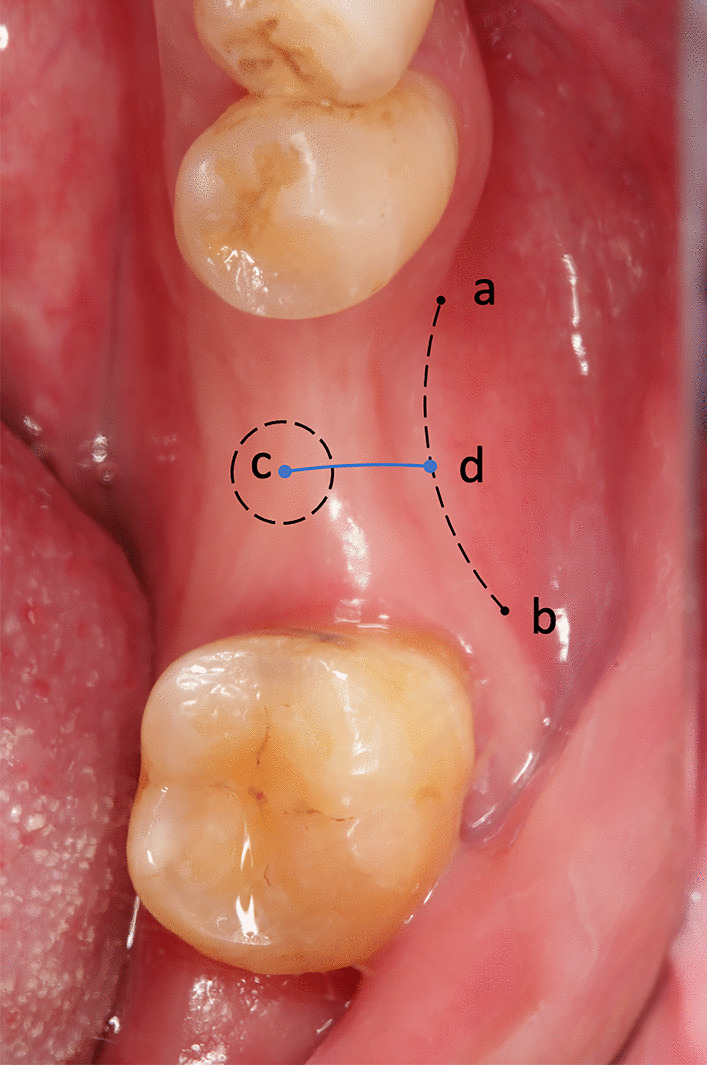
Representative measurement of KMW at the edentulous molar site. **a, b** buccal MGJ of the edentulous molar site; **c** center of the designed implant position; **d**: the point at which the vertical line from point c met the buccal MGJ; cd: buccal KMW

The clinical parameters of the edentulous molar sites and the adjacent teeth were recorded before the sites underwent implant treatment. The buccal KMW of the edentulous molar sites was measured in millimeters from the central point of the planned implant site to the buccal MGJ using a Williams periodontal probe (Fig. 1).

The following parameters of the adjacent teeth were also recorded:

1. Counting of the adjacent teeth: 0 = no existing adjacent teeth; 1 = 1 adjacent tooth existing at the mesial or distal side of the edentulous molar site; 2 = adjacent teeth existing at both sides.
2. GP: determined by the visibility of the outline of the Williams periodontal probe when placed into the gingival sulcus. According to Le et al. [31], GP was scored as 0 = thin (both the outline of the probe at the gingival margin and the tip of the probe could be recognized); 1 = medium (only the outline of the probe at the gingival margin could be recognized); and 2 = thick (both the outline of the probe at the gingival margin and the tip of the probe could not be recognized).
3. KGW: distance from the mid-buccal gingival margin to the MGJ.

### Sample size calculation

Power analysis was performed to calculate the sample size. Samples of at least 115 patients were needed to detect 27.0% edentulous molar sites with inadequate KMW (< 4 mm) [9]. The type I error rate was assumed to be 0.05, and the beta error was 0.1 in a bilateral contrast (1−*β* = 0.90).

### Intraexaminer reliability

All the clinical parameters were determined and recorded by an experienced periodontist (WH). To test the intra-examiner reliability, 20 individuals in the present study were randomly selected and clinically examined twice in 2 weeks. The intraclass correlation coefficient (ICC) value was 0.983.

### Statistical analysis

All the parameters were entered into Excel 2016 (Microsoft Corporation) and analyzed by SPSS 26.0 (IBM Corporation). After using the Shapiro-Wilk test to test the normality, the differences in KMW (mm) between groups of edentulous molar sites which were divided by various parameters were analyzed by the paired Student’s *t-test* and one-way ANOVA. When the edentulous site had adjacent teeth at both mesial and distal sides, the GP and KGW of two adjacent teeth were generated to an average value for the data analysis [32]. The edentulous molar sites with 1 or 2 adjacent teeth were included in the analysis of the associations between their KMWs and the GP/KGWs of the adjacent teeth. To control for the confounding factor that more than one edentulous molar site in one subject was recruited, generalized estimation equations (GEE) and linear regression analysis were both used to explore the associated factors of buccal KM at edentulous molar sites. The significance level was defined as *α* = 0.05.

## Results

After preliminary screening, 165 individuals with 246 edentulous sites were selected. Ten individuals with 17 molar sites were excluded due to incomplete dental records, and 5 individuals with 7 molar sites were excluded because they received free gingival graft (FGG) before implant placement. A total of 222 edentulous molar sites in 150 patients (90 males and 60 females) aged 25 to 73 years with a mean age of 51.2 years were finally enrolled in this study. Each patient had 1 to 3 edentulous molar sites included and analyzed. Eighty sites were in the maxilla, and 142 sites were in the mandible. The smoking status of the patients and reasons for tooth loss/extraction are listed in Table 1.

**Table 1:** Demographics of patients and edentulous molar sites

	*N* (%)
Age (year)	
≤ 40	19 (12.7)
41–50	43 (28.7)
51–60	66 (44.0)
≥ 60	22 (14.7)
Gender	
Male	90 (60.0)
Female	60 (40.0)
Smoking status	
Smoker	33 (22.0)
Non-smoker	117 (78.0)
Tooth position	
Maxillary 1st molar	67 (30.2)
Maxillary 2nd molar	13 (5.9)
Mandibular 1st molar	84 (37.8)
Mandibular 2nd molar	58 (26.1)
Reasons for tooth loss	
Periodontal	124 (55.9)
Non-periodontal	98 (44.1)

Among the 222 edentulous molar sites, 24 sites had no adjacent teeth, and 198 sites had 1 or 2 adjacent teeth (Table 2). The GPs of adjacent teeth were mostly medium or thick, and the average KGW of the adjacent teeth at 198 edentulous sites was 3.67 ± 1.29 mm.

**Table 2:** Clinical parameters of adjacent teeth at edentulous molar sites

	*N* (%)
Score of adjacent teeth	
0	24 (10.8)
1	106 (47.7)
2	92 (41.5)
GP	
0 ≤ GP ≤ 1	40 (20.2)
1 < GP ≤ 2	158 (79.8)
KGW(mm)	
< 2	6 (3.0)
≥ 2	192 (97.0)

**Fig. 2:**
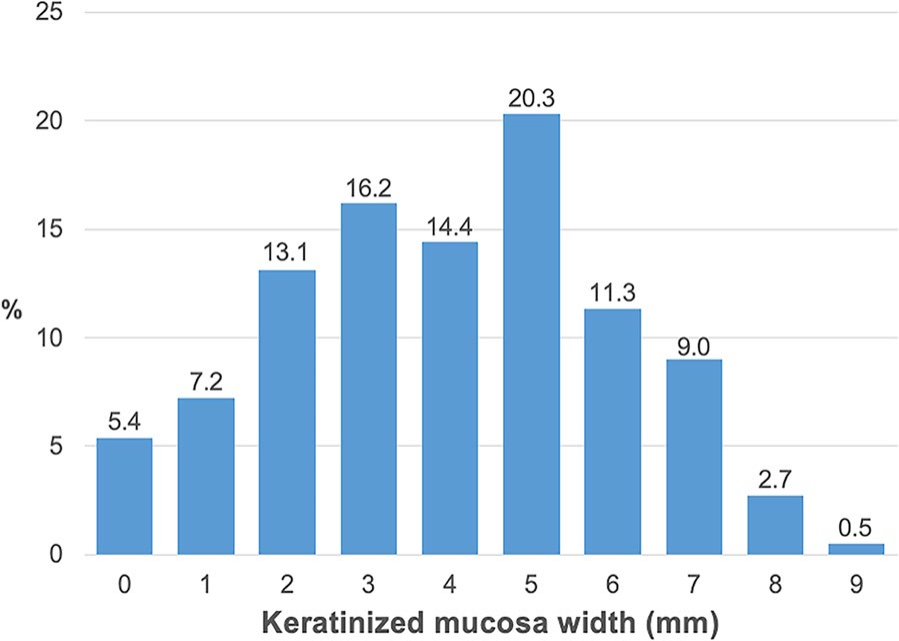
KMW at edentulous molar sites

**Fig. 3:**
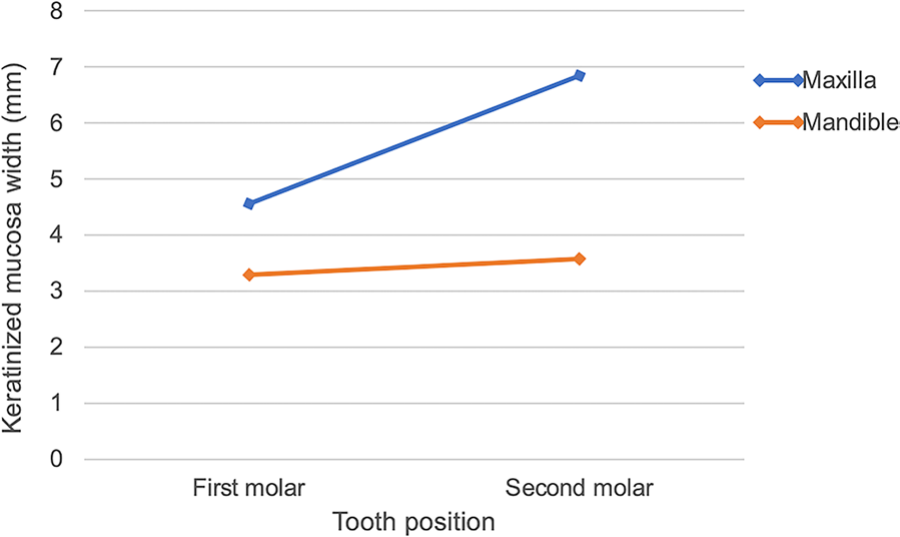
The average KMW at maxillary and mandible edentulous molar sites

**Fig. 4:**
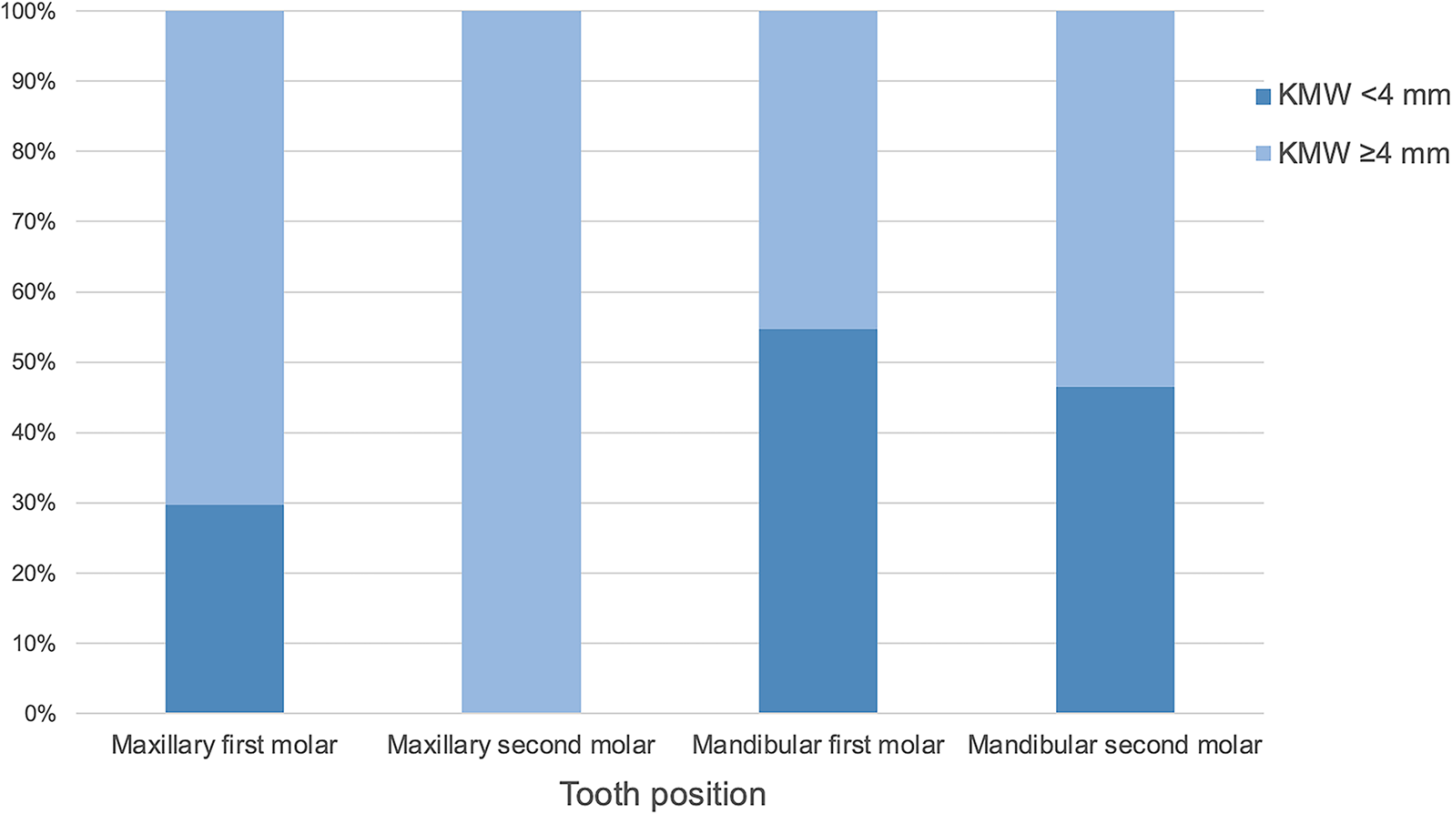
Distribution of the keratinized mucosa width at different tooth positions

The average KMW of 222 edentulous molar sites was 3.97 ± 2.06 mm (range of 0 to 9 mm), and 41.9% of sites presented with buccal KMW < 4 mm (Fig. 2). Among sites at different locations, the buccal KMWs of edentulous maxillary first and second molar sites were 4.56 ± 1.99 mm and 6.85 ± 1.14 mm, while those of mandibular sites were 3.29 ± 1.94 mm and 3.58 ± 1.71 mm, respectively (Fig. 3). A buccal KMW of < 4 mm was most frequently observed at edentulous mandibular first molar sites (54.7%), and the rest were at mandibular second molar (46.5%), maxillary first molar (29.8%) and maxillary second molar (0.0%) sites (Fig. 4).

The KMWs of edentulous molar sites were compared between groups classified by various parameters at the patient and tooth levels (Table 3). The ANOVA analysis showed that KMW was not significantly different between various age groups of patients (*p* = 0.217). The differences in KMW in patients with different sexes and smoking statuses were not statistically significant (*p* > 0.05). For different locations, edentulous maxillary molar sites had an average KMW of 4.96 ± 2.05 mm, while the KMW of mandibular molar sites was 3.41 ± 1.85 mm, which was significantly lower (*p* < 0.001). There were no significant differences between KMWs at first or second molar sites in either the maxilla or mandible and different reasons for tooth loss/extraction (*p* > 0.05).

**Table 3:** Keratinized mucosa width (KMW, mm) at edentulous molar sites classified by parameters on patient and tooth/site level

Variables	KMW Mean ± SD	*p* value
Age(Year)		
≤ 40	4.35 ± 2.12	
41–50	3.88 ± 2.31	
51–60	4.15 ± 1.90	
≥ 60	3.38 ± 1.86	0.217
Gender		
Male	4.02 ± 2.03	
Female	3.88 ± 2.11	0.597
Smoking status		
Smoker	4.17 ± 1.92	
Non-smoker	3.90 ± 2.10	0.394
Jaw		
Maxilla	4.96 ± 2.05	
Mandible	3.41 ± 1.85	< 0.001*
Tooth position		
First molar	3.87 ± 2.06	
Second molar	4.18 ± 2.05	0.297
Reasons for tooth loss		
Periodontal	4.05 ± 1.91	
Non-periodontal	3.88 ± 2.24	0.630
Score of adjacent teeth		
0	4.00 ± 2.45	
1	3.72 ± 2.06	
2	4.25 ± 1.92	0.068
GP		
0 ≤ GP ≤ 1	3.30 ± 1.87	
1 < GP ≤ 2	4.13 ± 2.01	0.018*
KGW (mm)		
< 2	2.83 ± 1.72	
≥ 2	4.01 ± 2.01	0.160

GP, gingival phenotype; KGW, keratinized gingival width

*p < 0.05

Sites with different numbers of adjacent teeth had similar KMWs (*p* > 0.05). Among the 198 edentulous sites with 1 or 2 adjacent tooth/teeth, the KMW values were significantly higher when the adjacent teeth presented with 1 < GP ≤ 2 than when the adjacent teeth presented with 0 ≤ GP ≤ 1 (*p* = 0.018). When the KGW of the adjacent teeth was < 2 mm or ≥ 2 mm, the average KMW at edentulous sites was 2.83 ± 1.72 mm and 4.01 ± 2.01 mm, respectively, while the difference was not statistically significant (*p* = 0.160).

A GEE with linear models was used to assess the factors associated with the KMW at edentulous molar sites (Table 4). The results showed that the KMW tended to be lower at mandibular edentulous molar sites than at maxillary sites (*p* < 0.001). The KMW at edentulous maxillary first molar sites was higher than that at mandibular first and second molar sites and lower than that at maxillary second molar sites (*p* < 0.05). Adjacent teeth presenting with medium and thick GPs (GP = 1.5, *p* = 0.16; GP = 2, *p* = 0.020) and higher KGWs (*p* = 0.038) were associated with higher KMWs at edentulous molar sites. Patient age, sex, smoking status, reasons for tooth loss/extraction and the number of adjacent teeth were not significantly associated with the KMW at edentulous molar sites (*p* > 0.05).

**Table 4:** The generalized estimating equation analysis of factors associated with the keratinized mucosa width at edentulous molar sites

	*B*	*p* value
Age (year)	− 0.011	0.509
Gender (female/male)	− 0.152	0.625
Smoking status (smoker/non-smoker)	0.265	0.489
Jaw (mandible/maxilla)	− 1.547	< 0.001*
Tooth position		
Maxillary 2nd molar/ Maxillary 1st molar	2.249	< 0.001*
Mandibular 1st molar/ Maxillary 1st molar	− 1.229	< 0.001*
Mandibular 2nd molar/ Maxillary 1st molar	− 1.101	0.002*
Reasons for tooth loss (periodontal/non-periodontal)	0.134	0.688
Score of adjacent teeth		
1/0	− 0.274	0.525
2/0	0.250	0.590
GP		
0.5/0	− 0.083	0.942
1/0	0.767	0.246
1.5/0	1.650	0.016*
2/0	1.340	0.020*
KGW	0.251	0.038*

GP, gingival phenotype; KGW, keratinized gingiva width; *p < 0.05

**Fig. 5:**
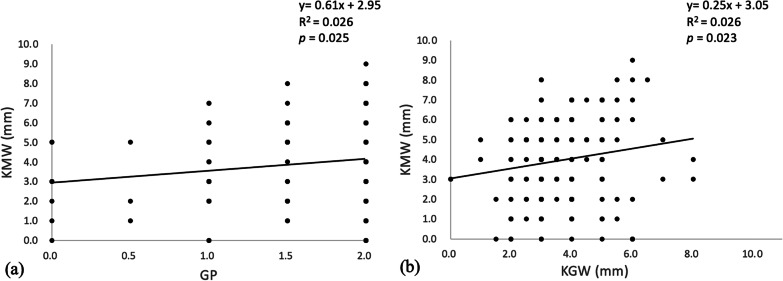
Linear regression analysis of the relationship between the KMW at edentulous molar sites and the GP and KGW of the adjacent teeth **a** KMW and GP. **b** KMW and KGW

Linear regression analysis was performed between the KMW of the edentulous molar sites and the GP and KGW of the adjacent teeth (Fig. 5). A significant linear correlation was found between KMW and GP (*n* = 198, r = 0.161, *p* = 0.025), and KGW was significantly correlated with KMW (*n* = 198, r = 0.161, *p* = 0.023). 

## Discussion

The positive impacts of an adequate KMW on peri-implant health have been reported in previous studies [33–35]. Therefore, assessment of the KMW at the edentulous ridge before implant placement is crucial during treatment planning for clinicians to predict the future peri-implant KMW and to predetermine whether soft tissue augmentation procedures are needed [14, 36]. However, to our knowledge, few investigations have focused on the dimensional characteristics of edentulous molar sites. The present study was the first to specifically evaluate the KMW of edentulous molar sites and explore the associated factors.

The findings of the current study revealed that the KMW at edentulous molar sites varied between different locations. The KMW of edentulous maxillary molar sites was significantly (1.5 mm) higher than that of mandibular sites (*p* < 0.001), which was similar to the studies evaluating the AGW and KGW around natural teeth [21–23]. In addition, the KMWs of the edentulous second molar sites were higher than those of the first molars in the maxilla, which was similar to the results reported by Endo et al. [23] and Lang and Löe [22]. It is speculated that the second molar sites were close to the tuberosity, which is covered by well-keratinized tissue and can be used as the donor site of keratinized tissue augmentation. This is the main reason why a higher KMW was discovered at edentulous second maxillary molar sites [37, 38].

Data from this study indicate that edentulous mandibular first molar sites were most frequently presented with inadequate KMWs (< 4 mm). Likewise, Roccuzzo et al. [12] pointed out that alveolar bone resorption is often accompanied by a decrease in KMW and reduced vestibular depth in the edentulous posterior mandible, for which the FGG procedure may be needed to facilitate oral hygiene procedures. Of particular note is that KMW < 4 mm was discovered at 41.9% edentulous molar sites, which reminded us that a relatively high percentage of dental implants may face a higher risk of lacking KMW after implant rehabilitation, especially at edentulous mandibular first molar sites. Thus, the demand for soft tissue augmentation should be considered carefully during implant treatment planning.

A positive correlation between the KMW of edentulous molar sites and the GP of the adjacent teeth was determined in this investigation, which was in accordance with previous studies evaluating natural teeth [24, 28, 29]. Reasons could be that the GP of the dentition may reflect the mucosal thickness of the edentulous sites; a thin GP is often related to an increased risk of gingival recession, which is also associated with a reduction in the KGW of edentulous sites [24, 28, 29].

The present study also discovered a positive correlation between the KMW at edentulous molar sites and the KGW of adjacent teeth, which was similar to the results of a cross-sectional study evaluating the peri-implant keratinized mucosa [4]. Thus, to achieve satisfactory biological and esthetic outcomes of implant reconstructions and the adjacent teeth, more attention and careful treatment planning are needed for the molars that are planned to be extracted and restored by implant restorations, as well as for edentulous molar sites with adjacent teeth surrounded by narrower KGWs.

One of the limitations of this study was the sample size. A larger sample size is needed to conduct a more convincing correlation analysis, since the sample size of some groups divided by various factors was significantly lower than that of other groups. In addition, the relationship between the KMW of the edentulous molar sites and the GT of adjacent teeth was not mentioned and should be assessed in a prospectively designed study. As the present study was designed retrospectively, the exact time of tooth loss was not collected, which may also have been a factor associated with the KMW of edentulous molar sites.

### Conclusion

Within the limitations, the KMW at edentulous molar sites was related to the location of tooth loss/extraction and the GP and KGW of the adjacent teeth. Future studies should analyze the changes in KMW before and after implant treatment, especially at mandibular molar sites or sites with a thin GP and a narrow KGW of the adjacent teeth.

## Data Availability

The datasets used and/or analyzed during the current study are available from the corresponding author on reasonable request.

## Abbreviations

KMW: Keratinized mucosa width
MGJ: Mucogingival junction
KGW: Keratinized gingiva width
AGW: Attached gingiva width
GP: Gingival phenotype
GT: Gingival thickness
ICC: Intraclass correlation coefficient
GEE: Generalized estimating equation
FGG: Free gingival graft
